# The HYP-RT Hypoxic Tumour Radiotherapy Algorithm and Accelerated Repopulation Dose per Fraction Study

**DOI:** 10.1155/2012/363564

**Published:** 2012-06-19

**Authors:** W. M. Harriss-Phillips, E. Bezak, E. Yeoh

**Affiliations:** ^1^School of Chemistry and Physics, University of Adelaide, Adelaide, SA 5005, Australia; ^2^Department of Medical Physics, Royal Adelaide Hospital, Adelaide, SA 5000, Australia; ^3^Department of Radiation Oncology, Royal Adelaide Hospital, Adelaide, SA 5000, Australia

## Abstract

The HYP-RT model simulates hypoxic tumour growth for head and neck cancer as well as radiotherapy and the effects of accelerated repopulation and reoxygenation. This report outlines algorithm design, parameterisation and the impact of accelerated repopulation on the increase in dose/fraction needed to control the extra cell propagation during accelerated repopulation. Cell kill probabilities are based on Linear Quadratic theory, with oxygenation levels and proliferative capacity influencing cell death. Hypoxia is modelled through oxygen level allocation based on pO_2_ histograms. Accelerated repopulation is modelled by increasing the stem cell symmetrical division probability, while the process of reoxygenation utilises randomised pO_2_ increments to the cell population after each treatment fraction. Propagation of 10^8^ tumour cells requires 5–30 minutes. Controlling the extra cell growth induced by accelerated repopulation requires a dose/fraction increase of 0.5–1.0 Gy, in agreement with published reports. The average reoxygenation pO_2_ increment of 3 mmHg per fraction results in full tumour reoxygenation after shrinkage to approximately 1 mm. HYP-RT is a computationally efficient model simulating tumour growth and radiotherapy, incorporating accelerated repopulation and reoxygenation. It may be used to explore cell kill outcomes during radiotherapy while varying key radiobiological and tumour specific parameters, such as the degree of hypoxia.

## 1. Introduction

Multiple studies have shown that hypoxia decreases cellular sensitivity to ionising radiation in living tissue. Consequently, there is an increase in radioresistance of hypoxic tumour cells following single or multifraction radiotherapy compared to oxic cells. Approximately 70% of locally advanced head and neck squamous cell carcinomas (HNSCC) have been reported to exhibit hypoxic regions, with median oxygen levels having a significant influence patient prognosis [1–3]. Reports from HNSCC clinical trials and experimental work commonly express hypoxia as the percentage of cells in the tumour having pO_2_ values less than 10, 5, or 2.5 mmHg, which is often very high (>50%) [4, 5]. In contrast, the average pO_2_ for healthy epithelial cells is approximately 40 mmHg [5].

 Tumour hypoxia occurs when the diffusion of oxygen from the surrounding tissue becomes insufficient in a nonvascularised tumour mass. It has been shown that tumours can grow up to a diameter of 1 to 2 mm without an independent blood supply [6, 7], after which neovascularisation is necessary for sustained growth. However, the new blood vessels may be chaotic in nature and possess faults such as holes and shunts. Consequently, an unstable and insufficient oxygen supply may develop causing tumour hypoxia. However, when a tumour is treated with fractionated radiotherapy, oxygen levels may begin to increase again during the process of tumour shrinkage, a phenomenon named reoxygenation (ROx).

 In aggressive tumours of epithelial origin such as HNSCC, cellular repopulation after trauma such high-dose irradiation, occurs through cell division of the surviving cell population. This repopulation can occur at an increased rate, a phenomenon named accelerated repopulation (AR). AR can have a detrimental impact on radiotherapy outcome, especially if the total treatment time is relatively long [8]. Multiple published HNSCC clinical trial reports conclude that the onset time of AR, or the so-called kick-off time, is between 2 and 5 weeks [8–12] after the start of treatment.

 As a supplement to clinical trials, Monte Carlo (MC) models can provide treatment response predictions which are (i) readily obtained and low in cost, (ii) reproducible, (iii) have the ability to account for the statistical nature of cellular kinetics and radiotherapy physics, and (iv) tumour specific depending on the data input into the model. MC methods and modern computing technology now make it possible to simulate the progression of individual tumour cells throughout the growth and treatment of a tumour approaching clinical sizes.

 The first reported computer model to employ MC methods was named CELLSIM by Donaghey and coworkers, published in the early 1980s [13]. One of the first models to include cellular-based stochastic methods as well as oxygen and nutrient diffusion factors came from work led by Dutching in the early 1980s and into the following decade [14–17]. Using their approach, a tumour up to 1 mm in diameter could be simulated and then treated with radiotherapy. Recent reports regarding stochastic hypoxic tumour modelling over that past two decades have come from work by group leaders such as Kocher, Titz, Borkenstein, and Stamatakos [18–24], in which the modelling of individual cells and hypoxia-related parameters have been applied.

 The *HYP-RT* model reported on here is based on the biological proliferative hierarchy of epithelial tissue to simulate oxic as well as hypoxic head and neck squamous cell carcinoma evolution. Cell division is tracked throughout growth and fractionated radiotherapy. *HYP-RT* takes into account the reoxygenation process of hypoxic tumours and the increased proliferation caused by accelerated repopulation. Stem cell symmetrical division is applied as the sole mechanism of AR, that is, the division of a stem cell into two daughter stem cells, based on reports of the dominance of this mechanism over other mechanisms such as cell cycle time shortening [8, 25]. A nonspatial approach in this probabilistic model means that cells are considered randomly placed within the tumour which is a justified approach considering that hypoxic tumour cells have been shown in multiple studies to be spatially irregular down to the submillimeter level, for example, in immunohistochemistry studies [26]. Compared to other models in the literature, *HYP-RT* has the benefits of fast computation, a high cell number, simple oxygenation data input in the form of a pO_2_ histogram, and the simulation of the combined effects of AR and ROx.

 The aim of the current modelling work was to extend the previous hypoxic tumour growth algorithm [27] and simulate conventionally fractionated radiotherapy. Improving the cell data storage and random cell selection aspects of the algorithm was also a goal, so that a full simulation could be completed in less than one hour. It was also important to model a sufficient cell number to surpass the approximate avascular exponential growth phase (10^6^ cells [28]) and reach a cell number approaching clinical levels, that is, 10^8^-10^9^ cells, while achieving statistically stable results (≤5%).

 This report outlines the methodology of the radiotherapy effect algorithm and discusses the key parameters of the model. Focus is placed on the ROx and AR modules of the algorithm and the effects of varying related parameters during simulations. The model is validated for the oxic tumour case through a comparison with linear quadratic (LQ) theory. The current work builds on a previous detailed description of the original hypoxic tumour growth algorithm [27] and the recently published conventional radiotherapy model outcomes [29] using the default parameters defined in the current report. In the following sections, modelling methods and algorithm design are detailed, along with the validation of modelling AR by means of increasing the stem cell symmetrical division probability and the consequential rise in dose per fraction needed to control the extra cell growth.

## 2. Methods

### 2.1. The Tumour Growth Algorithm

Carcinogenesis is initiated through cell division of a single oxic stem cell. Cell proliferation and subsequent tumour progression have been modelled by the continual division of cells into one or two viable daughter cells, with attributes allocated and saved to computer memory in an object vector array referred to as the *cellarray* The current data storage method differs from the two dimensional array methods previously reported. Methods were modified to allow for more efficient data storage that is 100% memory efficient at the time of tumour growth completion and enhanced efficiency relating to random sampling procedures. 

In the model, each element in the *cellarray* represents one cell *object* containing all cellular attributes as well as a *pointer* value indicating the position of the next chronological cell due to divide (the *linked list* method). MC methods are implemented to allow the random nature of the cellular kinetics and the effects of radiation treatment to be simulated using probability distributions. Cellular parameters, such as cell cycle time (CCT) and the cell type, resulting from mother cell division and the differentiation process, are allocated based on random number generation using uniform, normal, or exponential probability distributions and the Ziggurat random number generator [36].

Stem cells (*S*) first pass through the G0 quiescence phase with an exponential probability of duration and then enter the cell cycle. These cells are considered clonogenic and infinitely proliferating; however they may differentiate upon division or enter state of quiescence induced by low oxygenation. Other types of cells in the model include transit amplifying cells that cycle for a limited number of generations (*T* cells), differentiating cells (*D1 *and* D2 *cells), as well as fully differentiated cells (*D3* cells). Stem cells may divide into *S*, *T*, or *D1* cells, while transit cells may divide into *D1* or *D2* cells. In normal epithelium, the *D1* cells correspond to those created in the basal layer, while *D2* to those created above the basal layer. The percentages of each cell type in the model were verified as biologically plausible based on experiment epithelial tissue reports and other modelling studies [37–39].

 The parameter *Spercent* controls symmetrical stem cell division and represents the probability of a stem cell dividing into two daughter stems cells. This parameter was assigned a default value of 3.0%, which was based on achieving a total stem cell population of approximately 1% [27], a differentiated cell population of approximately 85% [37, 38], and an average tumour doubling time of 50 days [40]. Note that doubling times for tumour of differing oxygenation levels fell within a 35- to 65-day range.

 Due to the high percentage (85%) of noncycling cells required to ensure suitable tumour growth rates, the modelled tumours may be roughly equated to mid- to well-differentiated HNSCC disease. This is in contrast to poorly differentiated tumours. However, this distinction is difficult to quantify. The modelling of tumours exhibiting specific levels of differentiation was not a goal of the current work, nor was the interplay between the number of differentiated cells and tumour oxygenation status.

 The oxygenation of *oxic* tumours utilises a uniform pO_2_ distribution, ranging from 5 to 100 mmHg. These pO_2_ limits were set due to 5 mmHg often being used in published clinical trial reports as a hypoxic threshold and due to 100 mmHg [41] being the highest value measured experimentally in HNSCC Eppendorf studies. To model a biologically relevant range of tumour cell pO_2_ values for head and tumours, normalised data from Eppendorf studies [5, 41, 42] were implemented using a log-normal function and a random number algorithm written by J. Filliben (1982).

Tumour growth parameters values were set using biological data from the literature (e.g., oxygen distribution). If this was not possible, the model was used to explore the relative effects of certain parameters on other variables and parameters during the growth and treatment process (e.g., ROx increment size during radiotherapy). Key growth-related parameters and associated references are presented in Table 1.

To implement tumour hypoxia, a pO_2_ probability distribution is used to allocate values to daughter cells. For a mother cell producing only one cell, the mother cell pO_2_ is passed to the daughter cell. When two daughter cells are generated, one cell is randomly chosen to retain the mother cell pO_2_ and the other receives a new pO_2_ value from the distribution.

**Figure 1:**
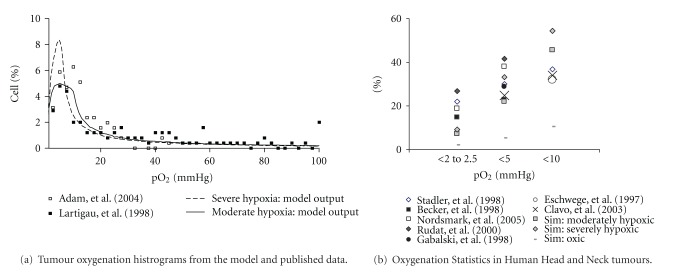
The distribution of tumour oxygen levels used (a) to simulate moderate and severe tumour and (b) in simulations (sim) compared to published data for three oxygenation ranges [4, 30–35]. The distributions in (a) represent the pO_2_ histogram outputs from the model using a log-normal random number generator.

 The first hypoxic pO_2_ distribution modelled is named moderate hypoxia. A second pO_2_ distribution representing a tumour with more severe hypoxia is also modelled. The severe hypoxia pO_2_ distribution is generated to achieve a relatively high number of cells in the low pO_2_ range (<10 mmHg) compared to moderate hypoxia and is tested in the model for the impact on tumour growth rate. Distributions with a higher (>3%) percentage of cells below 1 mmHg result in tumours that are too hypoxic and result in tumour shrinkage instead of growth. Both pO_2_ distributions along with published data are shown in Figure 1(a). Numeric histogram data for published versus modelled percentages of cells in different pO_2_ ranges are presented in Figure 1(b) and Table 2.

Cellular pO_2_ influences CCT [43, 48], implemented using an exponential function to slow the cell cycle with decreasing pO_2_. To account for the effects cell quiescence due to very low oxygenation, a threshold value of 1.0 mmHg is applied. These quiescent cells then die with a half life value of 4 days unless subsequently reoxygenated. As some cells have been shown experimentally to continue cycling even at very low oxygenation levels through anaerobic metabolism, only a percentage of cells with pO_2_ of less than 1 mmHg are made quiescent. The percentage for this parameter was determined by trial and error and ensuring that the total population of cells with this very low oxygenation corresponded with the log-normal pO_2_ distribution at the 0 to 1 mmHg level (3%).

 An analysis of the growth algorithm was performed to analyse the effects of the symmetrical stem cell division probability (*Spercent*) on tumour growth rate (doubling time, *T*_*D*_) and total growth time. This was carried out for oxic and hypoxic tumours, with the cell types in the population being validated. All of the current modelling work was programmed in the FORTRAN95 programming language within the Microsoft Visual Studio framework (2003).

### 2.2. The Radiotherapy Algorithm

 The radiotherapy algorithm was developed to simulate the effects of fractionated therapy, assuming that a uniform dose is delivered to all cells. LQ theory is used to define the average cycling cell survival probability using the standard surviving fraction (SF) equation based on alpha and beta parameters. This is calculated for each cell individually for each dose fraction in the schedule. For example, using alpha and beta values of 0.3 and 0.03, respectively (*α*/*β* = 10 Gy), and a standard 2 Gy per day treatment schedule, the SF value is 48.7%. However, this calculation is adjusted for the individual cell based on the cellular pO_2_. The adjustment is based on the oxygen enhancement ratio (OER) (1), which is implemented in the program by normalising the OER curve to a maximum value of one at 60 mmHg [49]:

(1) $$\text{OER}=\frac{1+0.81\left({\text{p}{\text{O}}_{2}}^{0.616}\right)}{\left(1+0.324\;{\text{p}{\text{O}}_{2}}^{0.616}\right)}.$$

**Figure 2:**
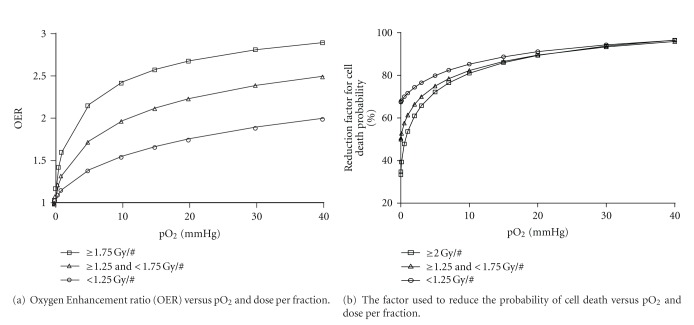
(a) Oxygen Enhancement Ratio (OER) curves implemented in the model for adjusting the radiosensitivity of cells during radiotherapy, based on cellular pO_2_ and dose per fraction, and (b) conversion of the OER curves into a probability of cell death factor, through OER curve normalisation.

For each fraction, all cells in the *cellarray* are chronologically assessed to determine if they will survive or die.  Figure 2 represents the relationship between (a) OER and pO_2_ (mmHg) and (b) probability of lethality and pO_2_ (mmHg) used in the model. Note that the effect of the actual dose being delivered is not shown in Figure 2, only the influence of pO_2_ on cell death when a specific dose per fraction is applied [50, 51].

 To model the gradual rise in tumour oxygenation during treatment, pO_2_ increments (3 mmHg) are distributed to the cell population at set time intervals. During reoxygenation events, a percentage of cells have their pO_2_ values increased by one or more pO_2_ increments, that is, by 0, 3, 6, 9, or 12 mmHg. Events are set to occur a few hours after each treatment fraction (default value of 4 hours [44]). The number of cells randomly chosen to receive the various increases in pO_2_ is based on Binomial theory (2):

(2) $$\begin{array}{c} P_{k}=\left(\begin{array}{c} n\\ k \end{array}\right)p^{k}{\left(1-p\right)}^{n-k}, \end{array}$$

where *n* is the total number of oxygen increments (equal to the number of cells in the population at the current time), *k* is the number of pO_2_ increments applied to a cell, and *P*_*k*_ is the probability of a cell receiving a *k* × 3 mmHg increase in pO_2_. The probability of five or more increments is below 0.5% and regarded as negligible.

 Default parameters in the ROx algorithm were set through observation of the rate at which hypoxia-induced quiescent cells were brought back into the cell cycle and the rate of change of the resulting pO_2_ histograms from the cycling cell population. Oxygen increment size was set by default to ensure that tumours shrink to between 10^5^ and 10^6^ cells (1 mm in diameter) with a final pO_2_ histogram resembling a uniform *oxic* distribution with all pO_2_ values ≥5 mmHg.

 Cells assigned to quiescence due to hypoxia (pO_2_ < 1 mmHg) have a probability of having their pO_2_ levels increased using a parameter to control the percentage of cells to be retrieved from the quiescent group and reentered into the *cellarray *that stores the cycling cells.

Accelerated repopulation is modelled by increasing the *Spercent* variable by a multiplicative factor (the *AR boost factor*) to simulate rapid tumour regrowth. As the range of possible onset times of AR varies in literature reports, a range of 0 to 3 weeks is analysed. This time range covers the possibility of immediate cell response as well as the latest onset time to have effect on total dose outcomes in the model.

**Figure 3:**
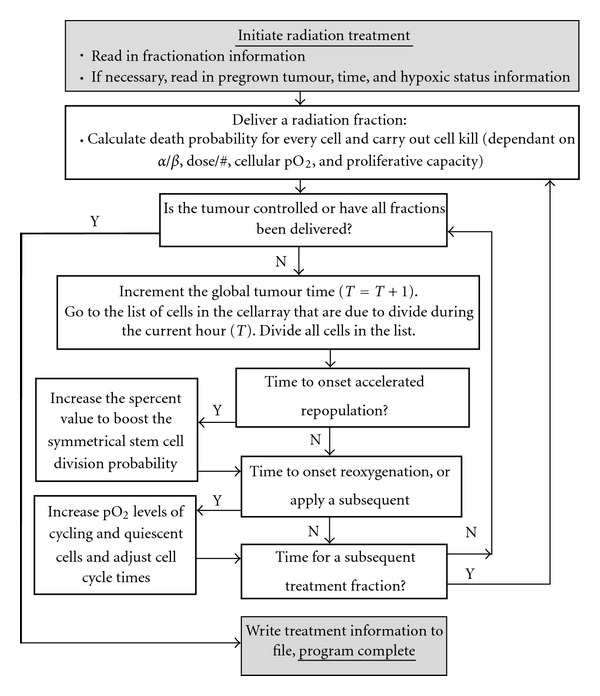
A flow diagram of the fractionated radiotherapy algorithm, where initiation of treatment is followed by continual cell proliferation between dose fractions.

 The default *AR boost factor* is based on a study of the extradose required to kill the cells that exist due to AR. When simulating no AR effects in oxic tumours 30 × 2 Gy (6 weeks of treatment) is required to control a tumour, therefore for all AR- and ROx-related simulations the effects of AR are calculated using 6 weeks at the iso-effect total treatment time. The duration of treatment for total cell kill was then compared to the 6-week standard time and the extradose per fraction required calculated for each simulation. Note that the extradose per fraction applies only during the weeks in which AR is occurring. The extra dose per fraction, *d*, is calculated using standard biological equivalent dose theory (3):

(3) $$\text{BED}=nd\left(1+\frac{d\alpha }{\beta }\right),$$

where BED is the total dose required from simulations to kill all cells after onset of AR, *α*/*β* =10 Gy, and *n* is the number of fractions for which AR is applied [52]. The default irradiation schedule used in the treatment module for this work is the conventional 2 Gy per day, 5 day per week dose schedule.  Figure 3 outlines the flow of the radiotherapy algorithm. The key parameters utilised in the model to simulate ROx and AR during radiotherapy are outlined in Table 3.

## 3. Results

All tumour growth simulation results apply to 10^8^ cell virtual tumours. The results summarise the impact of the *Spercent* parameter on growth rate and cell types percentage and verify that the modelling of hypoxia does not change the cell population structure, as intended. Treatment results relate to verification of the default *AR boost factor *for different AR onset times for both oxic and hypoxic tumours. Hypoxic tumour results are also presented for simulations varying the hypoxia-induced quiescent cell death half-life. Note that it may be possible for tumour cell of any proliferative capacity to avoid radiation cell kill due to hypoxia and become reoxygenated after treatment, contributing to local tumour recurrence. Moreover, hypoxia may cause mutations in the tumour cell population including dedifferentiation which may result in more aggressive tumour growth [60, 61]. Consequently, although survival of only the stem cells is traditionally considered to result in treatment failure, the number of fractions required to kill all stem, transit, and first-generation differentiating cells is presented.

 Hypoxic tumour radiotherapy results use the moderately hypoxic pO_2_ distribution shown in Figure 1(a). Severely hypoxic tumours with a higher number of cells in the 0 to 10 mmHg range were also modelled, but results did not differ significantly from moderately hypoxic results. This issue will be investigated in future work to discern the necessary change in the shape of the pO_2_ curve (peak width and peak height) required to obtain statistically different outcomes for very hypoxic tumours.

### 3.1. Tumour Growth Analysis and Algorithm Efficiency

**Figure 4:**
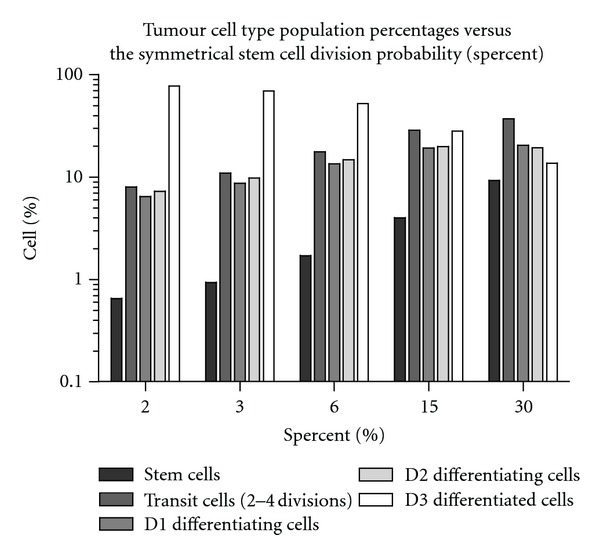
The average percentage of cell types within simulated tumours of 10^8^ cells, where (a) the symmetrical stem cell division probability parameter, *Spercent*, has been varied from 2 to 30% (standard deviation <1%).

**Figure 5:**
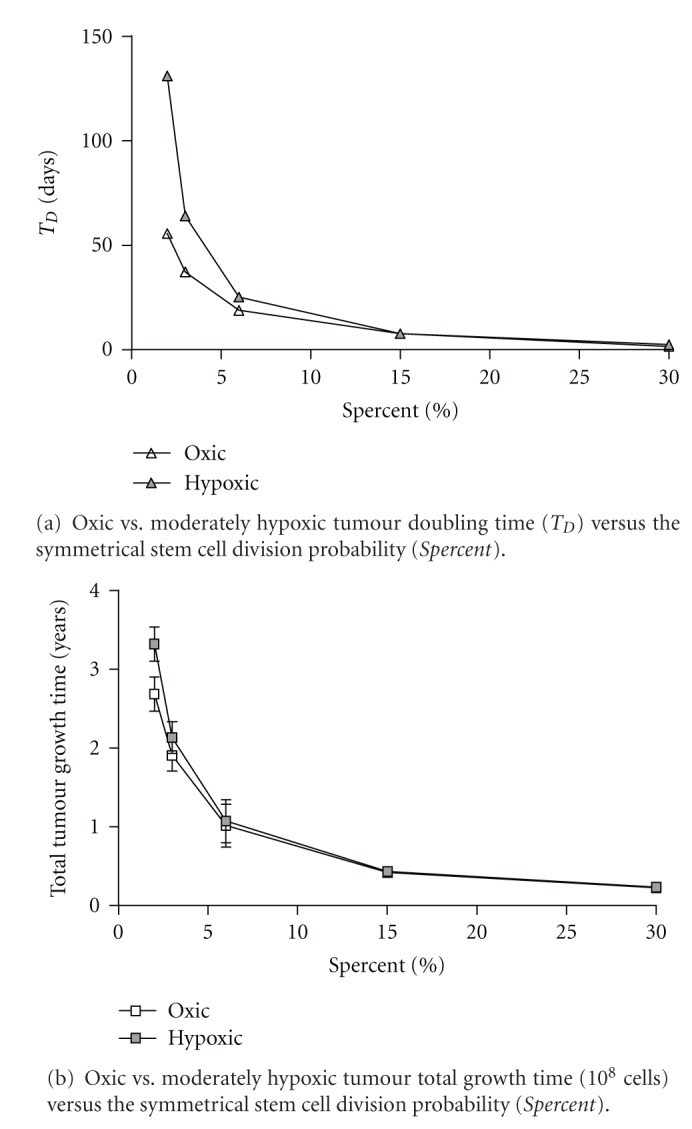
(a) The doubling time (*T_D_*), and (b) the total tumour growth times, versus the symmetrical stem cell division probability (*Spercent*) ranging from 2 to 30% for oxic and moderately hypoxic simulations of tumour growth up to 10^8^ cells. In Figure (a) the size of the error bars are negligible.

The constituent tumour cell population and the dependence of cell type percentage on the Spercent parameter are shown in Figure 4. The default value used for the Spercent parameter (3%) results in a distribution of cell types that closely match literature reports and a realistic statistically stable tumour growth rate after 10^4^ cells [38, 40, 62]. The tumour growth characteristics for oxic and hypoxic tumours are displayed in Figures 5(a) and 5(b). These tumour growth times (for a small 0.5 to 1.0 cm diameter tumour mass) agree with reported values of *T*_*D*_ for head and neck cancers [40]. Note that hypoxia-related parameters are set to maintain the distribution of cell types in the tumour throughout growth. Experimental data relating to how the cell percentages change when tumours are in a hypoxic state is difficult to obtain; therefore this effect has not been considered.

The *linked list* method of data storage allows for flexible and efficient cell data storage and enables 10^8^ cells to be propagated on a standard off-the-shelf computer. For oxic tumour growth simulations the average computation time is now less than fifteen minutes. For hypoxic tumours this may be extended up to thirty minutes. Total tumour growth times increase for hypoxic tumours due to the extracell death and therefore a longer time was required to propagate cells up to an equivalent tumour volume. Treatment-related parameters, especially the *Spercent* and *AR boost factor*, also alter the computation time because of their impact on stem cell exponential growth and the reduction in (non-cycling ) cells.

### 3.2. The Dependence of Cell Kill on the Alpha/Beta Ratio

**Figure 6:**
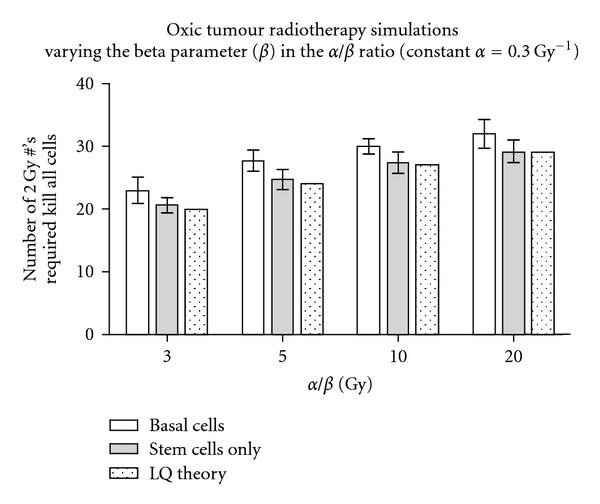
Comparison of the number of 2 Gy fractions required in the *HYP-RT* model to kill all basal or all stem cells compared to the linear quadratic (LQ) model (the first fraction that achieves <1.000 cells remaining), for oxic tumour conventional radiotherapy.

The default *α*/*β* value in the model is 10 Gy. However, results for a range of *α*/*β* values help to verify that the model predicts the same level of cell kill as LQ theory for oxic tumours (Figure 6). In the comparison, *α* values were held constant (0.3 Gy^−1^) while the beta value was varied (from 0.1 to 0.015 Gy***^−^*** ^2^), producing *α*/*β* values in the range of 3 to 20 Gy. For this analysis basal cell elimination as well as the elimination of stem cells only is considered. The stem cell simulation results are in good agreement with the LQ model, while results involving the cell kill of all basal cells are on average 2 to 3 fractions higher.

### 3.3. Oxic Tumour Radiotherapy

**Figure 7:**
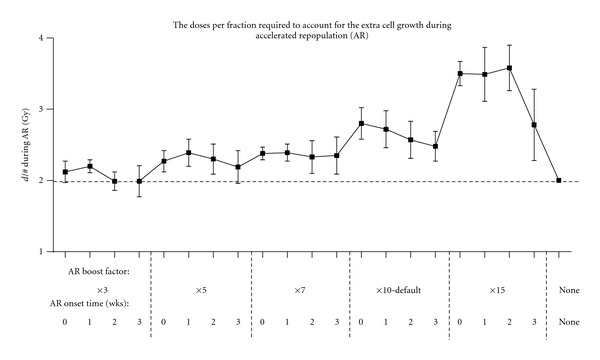
The increase in dose per fraction (*d*/#) required during conventional radiotherapy of oxic tumours to account for accelerated repopulation (AR), assuming a fixed total treatment time of 6 weeks and the increase in d/# coinciding with the onset of AR.

A factor is used in HYP-RT to increase the stem cell symmetrical division probability to model accelerated repopulation (the AR boost factor). To determine the most plausible AR boost factor, the onset times of AR are varied from 3% to 15% and from 0 to 3 weeks, respectively, and the consequential extra dose per fraction is required to account for the extracell growth calculated (Figure 7). Note that the increases in doses per fraction shown in Figure 7 apply during the period of AR only therefore the week of AR onset is not a critical parameter.

 An *AR boost factor* value of 10 results in an extra dose between 0.5 and 0.8 Gy per fraction, which is consistent with clinical trial reports [8, 63, 64]. An *AR boost factor* less than 10 increases the dose per fraction by 0.3 Gy or less, while an *AR boost factor* more than 10 results in an extra dose per fraction above 1.0 Gy. An *AR boost factor* of 10 was intuitively expected to impact on tumour response in a biologically plausible manner, as there have been reported increases in tumour growth rate of up to 10 times (reducing the potential doubling time (*T*_pot_) from approximately 10 to 20 days down to as low as 2 days [8, 65], approaching the stem cell division time.

 An *AR boost factor* of 10 decreases the tumour doubling time after the onset of AR; for example, for moderately hypoxic tumours the tumour doubling time decreases from 65 days down to 4.4 days after the onset of AR. Similarly for oxic tumours, doubling times decrease from 37 days down to 3.7 days after the onset of AR (all standard deviation errors <1 day). For moderately hypoxic tumours these doubling times have good agreement with HNSCC published data [8].

### 3.4. Hypoxic Tumour Radiotherapy

**Figure 8:**
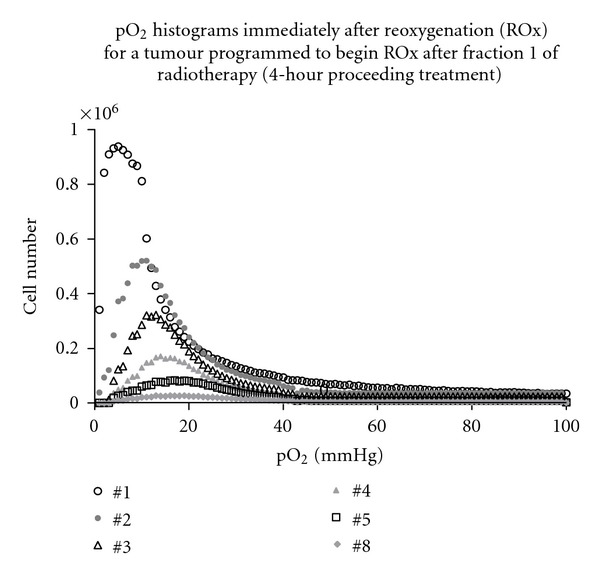
Oxygenation (pO_2_) histograms after each fraction of simulated conventional radiotherapy, ranging from fractions 1 to 8 of a moderately hypoxic tumour. In this example, treatment is initiated when the tumour population has reached 10^8^ cells and hypoxic quiescent cells are reoxygenated above 1 mmHg by fraction number 11 (5 × 10^6^ cells) and above 5 mmHg by fraction 20 (5 × 10^5^ cells).

 Interfraction pO_2_ histograms for a reoxygenating tumour are shown in Figure 8. ROx events are initiated in simulations after the first dose fraction in this example, with full ROx occurring by fraction 11. The model smoothly moves the peak of the histogram curve to the right-hand side, to higher average pO_2_ levels, simulating gradual ROx in the tumour, as desired.

**Figure 9:**
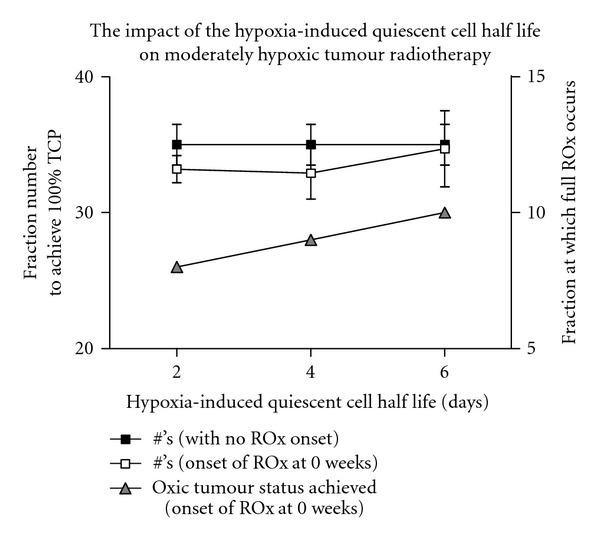
A comparison of the average number of conventional radiotherapy fractions with simulated reoxygenation (ROx) with no accelerated repopulation, required for cell kill of moderately hypoxic tumours when varying the half life of hypoxic quiescent cells.

 Cells exhibiting very low oxygenation (pO_2_ < 1 mmHg) levels enter a hypoxia-induced quiescent state. The modelled half-life of cells in this state does not impact significantly on the number of fractions required to control the tumour; however it does alter the timing of full ROx.  Figure 9 shows the impact of the hypoxic cell half life on cell kill and full ROx timing when varied from 2 to 6 days.

 The timing of ROx applied after completion of each fraction of conventional therapy was analysed for the impact on the total dose required to kill all cells. No significant difference was found between applying ROx either 4 or 23 hours (just preceding the next daily fraction) after a fraction. This result is expected since only conventional treatments were simulated in this study, with 24 hours between fractions. However, for future simulation work involving alternate schedules (hyperfractionated schedules with less than 2 Gy per fraction), this may change since ROx may occur during or after the next, same day fraction.

**Figure 10:**
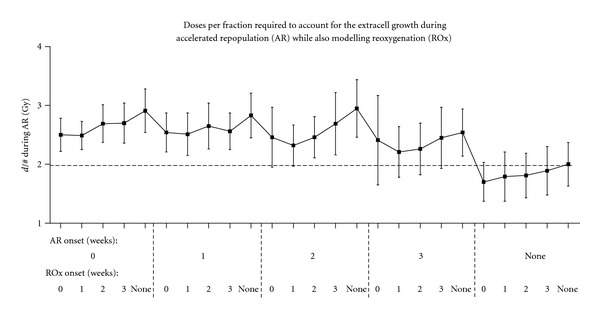
The increase in dose per fraction (*d*/#) required during conventional radiotherapy of moderately hypoxic tumours to account for accelerated repopulation (AR), assuming a fixed total treatment time of 8 weeks and the increase in *d*/# coinciding with the onset of AR, for various onset times of AR and ROx.

The effects of AR on the dose per fraction required to maintain total treatment times for hypoxic tumours were also studied. During these calculations, 8 weeks is used for the standard treatment time, as this is the treatment time required in hypoxic tumour simulations with no AR considered. The increase in dose per fraction (above the standard 2.0 Gy) is 0.5 to 0.9 Gy using an *AR boost factor* of 10 (Figure 10), closely matching oxic tumour results. The dose per fraction required during AR to control the extracell growth is relatively consistent for different AR onset times (as it is in the oxic tumour study), because the dose increase only applies *after* AR onset. With ROx simultaneously considered, the dose per fraction reduces slightly but is still approximately an extra 0.5 Gy per fraction.

An *AR boost factor* of 10 is considered the most valid value for this parameter based not only on the dose per fraction study but also according to the decrease in tumour doubling times predicted by the model; for example, the tumour doubling time reduced to 1 to 5 days after onset of AR compared with 35 to 65 days before onset of AR, with the range depending on oxygenation status.

 These results also indicate that the onset time of AR is likely to be ≤2 weeks if ROx occurs at ≤2 weeks. However, the onset time of AR could be >2 weeks if ROx also occurs late or not at all (based on dose per fraction increases within 0.5 to 1.0 Gy). Note that in all text and figures, error bars represent standard deviations based on nine simulations per parameter set. The statistical software package Prism 5 (v5.02, *GraphPad Software *Inc.) and Microsoft Excel 2003 were used in the analysis of the data.

## 4. Discussion

Onset times of ROx may be varied in the model from zero (immediately after treatment initiation) to three weeks. Immediate ROx is biologically possible because of the reduced demand of oxygen arising from the death of the first lethally hit cells (some hours after the first fraction). Variability of onset of ROx occurs because of other considerations such as the structure of the vascular and supporting tissues of the tumour and the dynamics of dead cell clearance from the tumour mass. All of these factors are likely to vary among patients, making the prediction of onset time of ROx for a particular tumour especially challenging. The timing of ROx events after each treatment fraction does not impact on the final cell kill results for the simulated conventional treatments in this study beyond the statistical error of the algorithm. The impact was not expected to be significant because all ROx events were programmed to occur before the next daily fraction.

 ROx is ideally modelled continuously throughout tumour shrinkage. Modelling increases to cellular pO_2_ levels at every hour of the radiotherapy regimen is computationally exhaustive; therefore as a compromise, ROx events are modelled after every daily dose fraction. Ideally, modelling of ROx would also be based on human pre- and midtreatment tumour oxygenation assay or imaging data, for example, PET, CT, MRI, or US imaging methods; however such data is not readily available for every patient. In the future more data of this kind may become available through research efforts including stratification of patients in clinical trials investigating tumour oxygenation dynamics. Trial outcomes would be very useful for radiobiological modelling of tumour treatment response; however they may never be able to predict individual tumour oxygenation behavior for a specific patient. At this stage, the model can provide quantitative information about the relative importance of hypoxia and reoxygenation during radiotherapy. Results highlight the need to pursue research into techniques for noninvasively and efficiently collecting individual tumour data for input into oxygenation specific models.

 Modelling the onset time of AR as early as zero weeks is based on the hypothesis that the microscopic response of tumour cell injury may start after the first radiative damage event. This somewhat contradicts reports based on clinical trial data of AR onset or kick-off times in the order of 2 to 5 weeks [8, 9, 11, 55, 65]. However, these reports are based on large patient averages of total treatment time effects and results of which are not necessarily representative of when AR is initiated at the microscopic level. Like ROx, AR onset times are likely to vary from patient to patient; thus for this study a range of onset times of 0 to 3 weeks is used.

 Due to the complexity of biological factors required in the model, it was necessary to make a number of assumptions/limitations. The reduction of the cell cycle time as an AR mechanism has been shown to have a significant but relatively small effect on tumour response; however this was not modelled. It is likely that a number of these mechanisms are induced together and are more significant if used in combination [8, 25, 65, 66]. Abortive division of sterilised stem cells (rather than differentiating) may also contribute to repopulation of tissue after irradiation [67]. However, these effects are not as well understood, with limited studies in the literature.

 The radiotherapy effect algorithm does not take into account repair of radiation-induced cell damage; however the modelling of cellular repair will be considered in future work. Spatial information of the tumour cells was not taken into account; however this was not a hindrance to the current results which are concerned with homogeneous dose delivery. For future spatial dose delivery application, such as IMRT or dose painting, the modelling of specific hypoxic subvolumes of hypoxia will be a necessary addition to the model.

*HYP-RT *modelling methods vary from other recent stochastic hypoxia and radiotherapy modelling methods, involving individual cells or cell groups [20, 24, 68], because the tumour oxygenation data required is simple and easily input in the form of a pO_2_ histogram. The pO_2_ histogram may also be manipulated during tumour growth (or treatment) if required to model dynamic oxygenation effects.

 Current issues with this model and other models of this kind include the requirement of data gained through methods that are invasive for the patient; however this is improving as imaging techniques and associated marker drugs are being researched and trialled.

 Possible current uses for the model include the study of cellar kinetic mechanisms and observations of (i) the relative differences in total doses required when AR and ROx are onset at various times, (ii) the differences in the total doses required for tumours of varying oxygenation levels, and (iii) the prediction of the effects of treatment breaks on the extradose required to compensate for the break.

## 5. Conclusion

Due to the complexities and dynamic nature of tumour oxygen and reoxygenation during radiotherapy, MC methods remain the most comprehensive and simplistic way of incorporating hypoxia into a tumour model. The *HYP-RT* model builds upon previous tumour growth modelling work and is capable of modelling radiation cell kill for tumours comprised of up to 10^8^ individual cells. Computational and temporal efficiency has been improved compared to previous model versions, with better use of memory space and more efficient selection and allocation of randomised parameters. Simulations of tumour growth and radiotherapy treatment can be performed in approximately thirty minutes or less. The way in which tumour hypoxia has been modelled is simple yet specific, enabling individual tumour data input in the form of a pretreatment pO_2_ histogram.

 The reoxygenation algorithm provides a method of gradually altering the initially hypoxic tumour oxygenation histogram throughout treatment, to model the process of oxygenation increase for a hypoxic tumour. The accelerated repopulation algorithm provides a method of increasing the cell propagation rate, using a parameter that increases the symmetrical stem cell division probability, with a factor of ten found to be most suitable value based on a study involving the increase in dose per fraction required to the kill the extracell growth during conventional radiotherapy.

 Future aims include efficiently modelling 10^9^ cell virtual tumours to provide an even larger individual cell-based model and the conversion of the code into the more modern C++ programming language. The model has already been used to explore the conventional radiotherapy schedule for hypoxic tumours [29] and will be reported on in the near future regarding alternate radiation regimens for tumours of different oxygenation levels.

## Figures and Tables

**Table 1:** Default parameter values and ranges available in the tumour growth algorithm.

Parameter	Default	References	Comments
*Cell total*	10^8^	N/A	The fully grown tumour cell limit.
*Spercent, *symmetrical stem cell division probability (%)	3%	N/A	This parameter was set to produce a 1% total stem cell population in the tumour.
Low oxygen limit for cell cycle arrest (mmHg)	1 mmHg	[43, 44]	At this pO_2_ level hypoxia-induced quiescence may be initiated.
Percentage of cells exiting the cell cycle (with pO_2_ <1 mmHg) (%)	50%	N/A	The total number of hypoxia-induced quiescent cells (<1 mmHg) = 3% using this parameter value, in line with the pO_2_ histogram used.
Tumour cell number threshold for hypoxia	10^6^	[6, 7, 28, 45]	Hypoxia is modelled after the cell number is reached by allocating pO_2_ from the modelled pO_2_ histogram.
Hypoxic cell half life due to necrosis: pO_2_ < 1 mmHg (days)	4 days	[46, 47]	Due to the 4- to 10-day hypoxic cell lifetime in human colon carcinoma spheroids, and 2 days in xenograft HNSCC.

**Table 2:** Tumour oxygenation histogram data for the three modelled oxygenation levels, indicating the modelled percentage of cells in four commonly reported pO_2_ ranges.

pO_2_ range (mmHg)	Oxic (%)	Moderately hypoxic (%)	Severely hypoxic (%)
0 to 2	2.1	7.3	9.4
0 to 5	5.2	22.1	33.2
0 to 10	10.4	45.6	54.5
0 to 20	20.8	65.4	69.6

**Table 3:** A list of model parameters used in the fractionated radiotherapy algorithm.

Parameter	Default	References	Comments
Accelerated repopulation (AR)—time of onset after initialisation during RT (weeks)	No onset	[8, 10–12, 25, 53–55]	The number of weeks into RT that AR is onset, 2–4 weeks observed in literature; however 0 week onset has been made possible for modelling microscopic response in a small tumour system.
Reoxygenation (ROx)—time of onset after initialisation during RT (weeks)	No onset	N/A	An extremely variable parameter and open to user input.
Time of ROx after a particular RT fraction (weeks)	4 hours	[44]	
*AR boost factor*	×10	[23, 25, 40]	The factor applied to increase the symmetrical stem cell division probability during AR.
ROx-induced incremental increases in pO_2_ (mmHg)	3 mmHg	N/A	The pO_2_ increment size during randomised reoxygenation after an RT fraction (linearly SF dependent), set to obtain full oxygenation by ~1 to 2 mm tumour diameter.
ROx percentage of the very low oxygenated cell population (%)	60%	N/A	The percentage of hypoxia-induced quiescent cells brought back into the cell cycle upon ROx after an RT fraction (linearly SF dependent), set to obtain full oxygenation when the tumour has reduced to 10^5^ to 10^6^ cells (from 10^8^ initial cells).
Alpha (LQ model) Gy^−1^	0.3	[56, 57]	Used in SF calculations (linear quadratic equation).
Beta (LQ model) Gy^−2^	0.03		Used in SF calculations (linear quadratic equation).
Noncycling cell radiosensitivity compared to oxic cycling cells	0.5	[58, 59]	Factor for the decreased radiosensitivity of noncycling cells, based on the likely increase in resistance of cells in resting phase (transit cells and stems cells however assumed to be equally radiosensitive in tumour cells).
